# Molecular Mechanisms Underlying Hepatocellular Carcinoma

**DOI:** 10.3390/v1030852

**Published:** 2009-11-09

**Authors:** Philippe Merle, Christian Trepo

**Affiliations:** 1 INSERM, U871, 69003 Lyon; Université Lyon 1, IFR62 Lyon-Est, 69008 Lyon, France; 2 Hospices Civils de Lyon, Hôtel Dieu, Service d’hépatologie et de gastroentérologie, 69002 Lyon, France; E-Mail: christian.trepo@inserm.fr

**Keywords:** hepatocellular carcinoma, stem cells, cellular stress, senescence, oncogenes, tumor suppressors

## Abstract

Hepatocarcinogenesis is a complex process that remains still partly understood. That might be explained by the multiplicity of etiologic factors, the genetic/epigenetic heterogeneity of tumors bulks and the ignorance of the liver cell types that give rise to tumorigenic cells that have stem cell-like properties. The DNA stress induced by hepatocyte turnover, inflammation and maybe early oncogenic pathway activation and sometimes viral factors, leads to DNA damage response which activates the key tumor suppressive checkpoints p53/p21^Cip1^ and p16^INK4a^/pRb responsible of cell cycle arrest and cellular senescence as reflected by the cirrhosis stage. Still obscure mechanisms, but maybe involving the Wnt signaling and Twist proteins, would allow pre-senescent hepatocytes to bypass senescence, acquire immortality by telomerase reactivation and get the last genetic/epigenetic hits necessary for cancerous transformation. Among some of the oncogenic pathways that might play key driving roles in hepatocarcinogenesis, c-myc and the Wnt/β-catenin signaling seem of particular interest. Finally, antiproliferative and apoptosis deficiencies involving TGF-β, Akt/PTEN, IGF2 pathways for instance are prerequisite for cancerous transformation. Of evidence, not only the transformed liver cell *per se* but the facilitating microenvironment is of fundamental importance for tumor bulk growth and metastasis.

## 1. Introduction

Hepatocellular carcinoma (HCC) is one of the most prevalent cancers worldwide, developing mainly in cirrhosis. Hepatitis B (HBV) or C virus (HCV) chronic infections account for 75% of HCCs whereas nonviral etiologies as alcohol, genetic or metabolic disorders represent less than 25% of cases. Furthermore, western countries suffer from a substantial and constant increase of HCC incidence due to HCV infection. Dramatically, HCC is a poor prognosis tumor, and is the first cause of death in cirrhotic patients. Current therapies are rather inefficient, mainly due to usually late diagnosis and high recurrence rates within the remaining cirrhotic liver after surgical resection [1–3].

Hepatocarcinogenesis is tightly linked to chronic liver damage, and rarely develops in healthy liver. That might be due to the possible requirement of chronic inflammation and cell divisions in a context of cellular stress which lead towards the step-wise acquisition of genetic and epigenetic hits necessary for cellular transformation. In addition, the virus persistence *per se* can trigger deregulation of the cellular machinery. By contrast to HCV, HBV can integrate into the host genome, leading to genomic instability, rearrangements and more rarely *cis*- or *trans*- activation of proto-oncogenes. Although the direct involvement of viral proteins in hepatocarcinogenesis is not clear, it seems that HBx and Pre-S2 for HBV as well as core and others for HCV can interact with and deregulate cellular machinery. However, data was obtained from *in vitro* transfection assays or *in vivo* transgenic mouse models. Nevertheless, these models support supra-natural viral protein expression levels, and nothing is known about these interplays in a context of natural viral infection [4–6].

Herein we will aim to describe the general mechanisms which could be involved in hepatocarcinogenesis independently of etiologic factors underlying the chronic liver disease.

## 2. Tumor Bulk and Cancer Stem Cell Concept

**Figure 1.**
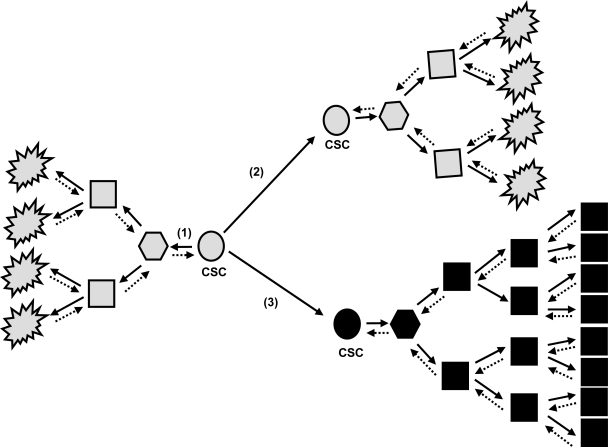
The cancer stem cells (CSC) model. Proposed hierarchical organization of a malignant clone. CSC have self-renew and tumorigenic capacities. They can give rise to more and more differentiated cancerous cells which the last ones in the hierarchical organization may have low or absent proliferative capabilities (1). During asymetric division at self-renew, the original CSC can give birth to the identical CSC (2) that will lead to the same tumor clone (grey), or can give birth to a genetically/epigenetically modified CSC (3) that will lead to a more aggressive tumor clone (black) with differentiation blockage and higher proliferative capabilities. To date, it is not clear whether the more differentiated cells can revert back and regain a more stem cell properties. As appreciated from the definition of a CSC, this cell is not necessarily derived from a normal tissue stem cell. Alternatively, hypothetical genetic/epigenetic changes caused in more committed and differentiated cancerous cells might be very well involved in mechanisms allowing these cells to acquire cancer stem cell capabilities.

The most common and unifying condition associated with hepatocarcinogenesis is cirrhosis, which develops after long latencies (20–40 years) of chronic liver disease. HCC risk remains low during chronic liver disease but dramatically increases at the cirrhotic stage. Hepatocarcinogenesis remains partly obscure. Initially, a variety of genetic and epigenetic alterations have been detected in human and experimental HCCs. Later on, DNA microarray analysis has led to an extensive integrative approach, leading to identification of clusters of HCCs that allow comparison between phenotypes in experimental and human HCCs, and may predict outcome of patients. However, none of the identified genes is universally expressed by tumor cells that are heterogeneous in their morphology, clinical behaviour, and molecular profiles in the tumor bulk [7–9]. These observations lead to the suspicion that the current studies might have focused only on the heterogeneous “end products” – *i.e.* “adult” tumor cells within the tumor bulk - but not the “root” cell (Figure 1) [10–13]. More interestingly, molecular signatures from cirrhotic nonneoplastic tissues can predict occurrence/reccurrence of HCC [14], letting hypothesize that although “root” cells exist within the heterogeneous “end product” tumor bulk, they also might be present in cirrhotic tissues prone to develop HCC.

Tumors originate from normal cells as a result of accumulated genetic/epigenetic changes. It was initially believed that cancers arose exclusively by de-differentiation of mature cells, and tumor cell heterogeneity could be explained by the clonal evolution model [15]. More recent findings suggest that heterogeneity may come from derivation of endogenous progenitor/stem cells or de-differentiation of a transformed cell [16–17]. This hypothesis supports an early proposal that cancers represent “blocked ontogeny” and a derivative that cancers are transformed stem cells (Figure 1). The normal liver has tissue-determined stem cells and ductal bipotent committed progenitors that are scarce and not brought into play except with the occurrence of both liver injury and inhibition of proliferation of mature hepatocytes. These latter are not terminally differentiated and can respond to injury by highly regulated proliferation. Although the cell type giving rise to HCC has been shown as dependent on many factors in experimental hepatocarcinogenesis, few is known in humans. One can speculate that “root” cells might come from the neoplastic transformation of different normal liver cell types: periductal stem cells, bipolar ductal committed progenitor cells, or differentiated hepatocytes. Thus, it would not be surprising to find HCC as arising differentially from one (or several) of them, depending on extrinsic factors such as viral infection, or deregulation of intrinsic key pathways [18–23]. The maturation arrest of cells at various stages of differentiation in a hierarchical cell lineage may best explain the various types of human liver cancer. From analysis of established HCCs, we might speculate that HCCs contain cancer stem cells (CSC) – *i.e.* cells with stem-cell-like properties of immortality, resistance to therapy, and transplantability. As hepatocarcinogenesis is likely a dynamic process leading from a normal cell towards an initiated root cell and thereafter a hugely heterogeneous tumor bulk, abnormalities useful for initiation of root cells are not mandatory found in all cancerous cells forming the tumor bulk. In addition, abnormalities found in tumor bulks might evolve with time and/or under pressure of anti-cancer therapies, thus strengthening the need of cautious when interpreting a molecular profile. However, some fundamental events have been described as key steps in cellular transformation and very likely necessary and sufficient to allow each cell to get and keep the cancerous phenotype.

## 3. Oncogenic Stress and Cellular Behaviour

**Figure 2.**
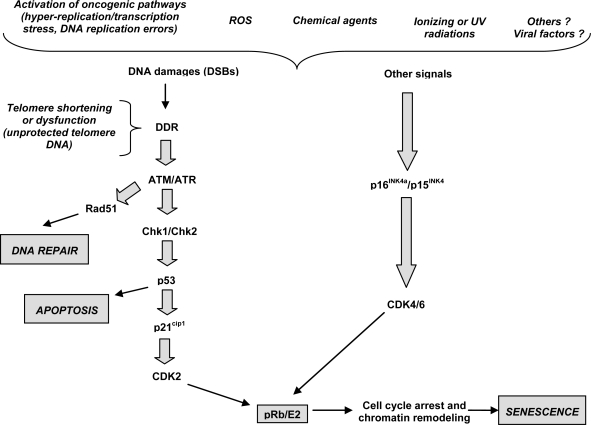
The proposed molecular mechanisms of cellular senescence. Oncogenic stress result in DNA damages as double strand breaks (DSBs) that lead to activation of ATM/ATR kinases. This leads to stabilization of the Rad51 repair protein needed for DNA DSB repair. Besides DNA repair induction, cooperative action of ATM/ATR, ARF and Chk1/Chk2 and p53 is crucial for the induction of either apoptosis or cell cycle arrest and induction of senescence. Another possibility is that other factors such as reactive oxygen species (ROS) or other physical, chemical of viral agents could induce the accumulation of DNA damages or activate, by alternative mechanisms, the p15^INK4b^/p16^INK4a^ cascade. All known senescence pathways converge at the level of activation of CDKIs (p15^INK4b^, p16^INK4a^ and p21^Cip1^) that keep the pRb protein under its active form. The pRb inhibits E2F and prevents the expression of growth-promoting genes for cell cycle exit. Furthermore, pRb recruits growth-promoting genes into a facultative chromatin structure for permanent silencing and growth arrest.

Cancer cells contain multiple genetic/epigenetic alterations, and chromosomal aberrations. It has been rationalized that a long period of time is required for any individual cell to accumulate the right combination of alterations that promote the cancer cell phenotype. Alterations consistently found in cancer cells are selected because they confer a growth advantage by either activating growth promoting pathways, inactivating growth inhibitory cascades or allowing alterations to accumulate [24]. Over the life span any individual cell can acquire multiple alterations with oncogenic potential, yet only a fraction of them will experience cancer transformation. This fact suggests that organisms evolved mechanisms to prevent oncogenic transformation, the so called anti-oncogenes or tumor suppressor genes. Tumor suppressors may avert cancer by preventing alterations, inducing cell death or a program of cell division arrest known as cellular senescence. This senescence has been experimentally modeled by enforcing the expression of oncogenes in primary cells. The astonishing outcome of these manipulations is that oncogenes trigger antiproliferative responses preventing progression to malignant transformation. These responses bring to an end-proliferation due to cell death or a permanent cell cycle arrest called senescence (Figure 2). On a general point of view, and although it remains to be validated in hepatocarcinogenesis, these results imply mechanisms of DNA damage in cells expressing oncogenes, that may be secondary to reactive oxygen species and/or some form of “oncogenic stress” that affect normal DNA replication. Interestingly, DNA damage signals persist in cells that escape from senescence and go ahead towards cancer transformation [25].

### 3.1. Senescence pathways as tumor suppressor mechanisms in chronic liver disease

In healthy adult livers, hepatocytes are quiescent cells, being renewed slowly, approximately once a year. However, the liver has an extremely powerful regenerative capacity, as demonstrated experimentally in rodents, and as observed in patients with chronic liver diseases [26]. This regenerative capacity is due mostly to the ability of mature hepatocytes to proliferate in response to a diminution of the total liver mass either experimentally, or following exposure to viral and nonviral hepatotoxic agents. In addition, the adult liver seems to harbor hepatocyte-progenitor cells (< 0.1% of total hepatocyte mass) that are able to restore liver hepatocyte populations [27]. However, hepatocytes do not have unlimited replicative capacity, due to the lack of telomerase activity that is needed to avoid telomere shortening during successive cell divisions. This is best exemplified by decreased hepatocyte proliferation in the cirrhosis stage of chronic liver diseases, providing *in vivo* evidence for the exhaustion of hepatocyte proliferation capacity [28].

Senescence mechanisms in hepatocytes and in liver tissue are not well known. However, a limited number of *in vitro* studies with hepatocytes, as well as numerous descriptive *in vivo* studies in liver tissue provide sufficient evidence that hepatocytes can undergo senescence type changes. Limited proliferative capacity of somatic cells is controlled by replicative senescence. By contrast to primary hepatocytes which do not proliferate in culture, fetal hepatocytes display better proliferation capacity and can enter replicative senescence [29]. This is accompanied by progressive shortening of telomeres in a context of telomerase-free activity. In contrast to *in vitro* studies, *in vivo* senescence of human hepatocytes is better known. Replicative senescence displays a gradual increase from 10% in normal liver, to more than 80% in cirrhosis, being detected in 60% HCCs [30]. Telomere shortening during aging is slow and stabilizes at mid age in healthy liver, so that the loss of telomeric DNA does not reach a level to induce telomere dysfunction and DNA damage response (DDR). On the other hand, telomere loss is accelerated in chronic liver disease to reach lowest levels in the cirrhotic liver. Therefore, one plausible mechanism involved in cirrhosis is probably telomere-dependent senescence, the so-called replicative senescence [31].

Hepatocyte senescence that is observed in severe chronic liver diseases may also be induced by telomere-independent mechanisms, such as ROS-induced senescence (RIS) and oncogene-induced senescence (OIS). RIS and OIS are rare events under normal physiological conditions, but could more commonly occur in context of ROS overproduction and/or oncogenic signals activation, both leading to DDR and activation of the ATM/Chk/p53 pathway and, by alternative mechanisms, the p16^INK4a^/pRb pathway. OIS is a DNA damage response triggered by DNA hyper-replication [32], and several oncogenic pathways – *i.e.* activated Ras, c-myc or Wnt/β-catenin - have been shown as potentially involved in OIS in mammary cells or fibroblasts, but nothing is known about liver cell types [33–34]. As witness of DDR occurrence in chronic liver diseases, upregulation of DNA repair enzymes which may reflect increased DNA damages, has been reported in cirrhosis [35]. ROS production can be favored by the context of chronic liver injury with inflammation, cell death, oxidative stress as well as some of the etiological factors such as HCV and alcohol induce mitochondrial dysfunction [36]. Later on, we will describe the main oncogenic pathways found as activated in HCC and that might lead to OIS although this phenomenon of senescence-induction/senescence-escape has not been clearly demonstrated so far in liver cells during hepatocarcinogenesis.

### 3.2. Senescence-related aberrations in chronic liver disease and hepatocarcinogenesis

**Figure 3.**
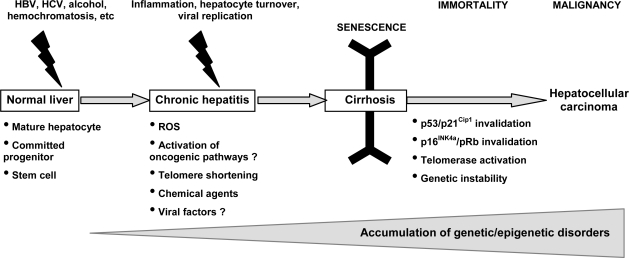
The proposed model of hepatocarcinogenesis. One or several types of liver cells (mature, hepatocytes, progenitor/stem cells) could support the multi-step process of accumulating genetic/epigenetic disorders leading to senescence, and subsequently bypassing senescence to reach cancer. Cirrhosis is a senescent state that could be induced by different factors such as ROS, oncogene activation and telomere shortening for instance. The senescent state is kept active by the p53/p21 and p16/pRb checkpoints. When these gatekeepers become inactive, the cell can bypass senescence, and re-enter cell cycle progression and DNA hyper-replication in a context of immortality, due to telomerase reactivation, and genetic insability. Thus, the cell is prone to acquire the last genetic/epigenetic hits necessary to get tumorigenicity and cancer stem cell capabilities.

The abrogation of DNA damage checkpoints could represent a selective advantage allowing clonal expansion of genetically altered hepatocytes at the stage of senescence (Figure 3). As stated earlier, the p16^INK4a^/pRb and p53/p21^Cip1^ pathways play crucial roles in senescence arrest as observed in different *in vitro* and *in vivo* models [37]. Thus their invalidation could help the initiated cells to escape the senescent process and go ahead to genetic instability and cancerous transformation. Strikingly, etiologic factors *per se* of HCC, such the viral HBV X protein, have been shown to help cells to bypass OIS [38].

The p53 pathway is a major tumor-suppressor pathway that: i) limits cell survival and proliferation (replicative senescence) in response to telomere shortening; ii) induces cell-cycle arrest in response to oncogene activation (OIS); and iii) protects genome integrity. The p53 pathway is affected at multiple levels in human HCC as follows: i) p53 mutations occur in aflatoxin-induced HCC (50%) and with lower frequency (20%–30%) in HCC not associated with aflatoxin; ii) microdeletions of p14^ARF^ occur in 15%–20% of human HCC but rarely in p53 mutant HCC; iii) increased Mdm2 expression has been observed in human HCC; iv) the vast majority of human HCC overexpresses gankyrin, which inhibits both the pRb and p53-checkpoint functions [39]. It seems likely that loss of p53 checkpoint function at the cirrhosis stage would lead to an expansion of hepatocytes with dysfunctional telomeres, chromosomal instability (CIN), and initiation of HCC. The p21^Cip1^ gene, downstream target of p53, is accumulated in cirrhosis by comparison to normal liver tissues [40]. Although p21^Cip1^ gene has not been found mutated in HCCs, its promoter is highly methylated in HCCs as compared to cirrhosis, showing that p21^Cip1^ is repressed in HCCs [41]. In addition to its effect on p53 checkpoint function, deletion of p14^ARF^ down-regulates expression of the *p27* tumor suppressor [42].

The p16/pRb checkpoint is another major pathway limiting cell proliferation in response to telomere shortening, DNA damage, and oncogene activation. In human HCC, retinoblastoma pathway alterations (p16^INK4a^, p15^INK4b^ or RB1 genes) – *i.e.* mutation/deletion of RB1 and/or methylation of p16^INK4a^, p15^INK4b^ promoters - are observed in more than 80% of cases, with repression of p16^INK4a^ by promoter methylation being the most frequent alteration [43], as well as gankyrin expression [39], indicating that the pRb checkpoint is dysfunctional in the vast majority of human HCCs. It is conceivable that an impairment of the pRb checkpoint would allow an expansion of hepatocytes with dysfunctional telomeres at the cirrhosis stage. In agreement with this assumption, p16^INK4a^ accumulates in cirrhosis by comparison to normal liver tissues, but p16^INK4a^ expression is diminished in premalignant liver tumors (small cell changes) and HCC [40].

Activation of telomerase occurs during the transition from premalignant lesions to HCC. More than 90% of human HCC show an activation of telomerase, which is the rate-limiting step for initiation of cell immortalization [44]. Experimental data from mice have shown that telomere shortening increases the initiation of liver tumors but telomerase deficiency limits the progression of early lesions toward macroscopic tumors [45]. A current hypothesis indicates that telomerase activation is necessary to limit telomere dysfunction and to prevent the accumulation of excessive levels of chromosomal instability and DNA damage that would impair tumor growth independent of p53 checkpoint function [46–47]. The molecular mechanisms involved in TERT suppression in somatic cells and its reactivation in cancer cells are poorly known. The integration of HBV DNA sequences into TERT gene provides evidence for a virus-induced deregulation of TERT expression, but this appears to rarely occur [48]. In addition, the viral X and PreS2 HBV proteins as well as the core HCV protein may upregulate telomerase activity [49]. Another hypothesis would be that HCC arise from stem/progenitor cell-like root cells that may already express TERT at sufficient levels to maintain telomere integrity. Experimentally, telomerase deletion limits the progression of p53-mutant HCCs with short telomeres [47]. These observations suggest that the aberrations affecting telomerase activity and senescence controlling genes such as p53 may cooperate during hepatocarcinogenesis.

In summary, HCC is characterized by mutational inactivation of p53, a major player in DNA damage-induced senescence. In addition, p15^INK4b^, p16^INK4a^, p21^Cip1^ CDKIs are often inactivated in this cancer mostly by epigenetic mechanisms involving promoter methylation. These changes may play a critical role in the bypass of senescence that is observed in most cirrhosis cases, allowing some initiated cells to escape senescence control and proliferate. Furthermore, it has been shown that Twist-1 and Twist-2 proteins can switch pre-senescent mammary cells towards epithelio-mesenchymal transition (EMT) and senescence-bypassing rather than towards senescence [34,50–52]. Both proteins override oncogene-induced premature senescence by abrogating the p53 and pRb pathways, and this phenomenon remains to be confirmed in hepatocarcinogenesis. In the absence of telomerase activity such cells would probably not survive due to telomere loss. However, since more than 80% of HCCs display telomerase activity, it is highly likely that the telomerase reactivation, together with the inactivation of major CDKIs, plays a critical role in HCC development by conferring premalignant or malignant cells the ability to proliferate indefinitely. However, cellular immortality is not sufficient for full malignancy. Thus, senescence-related aberrations that are observed in HCC cells, may confer a partial survival advantage that would need to be complemented by other genetic or epigenetic alterations to reach the cancerous phenotype.

## 4. Activation of Oncogenic Pathways

Hepatocarcinogenesis is multifactorial and various oncogenes, tumor-suppressor genes, growth factor genes and virologic factors are implicated [53]. Activation of oncogenic pathways in human HCC appears to be more heterogeneous compared with other cancer types. It remains largely unknown the exact sequence of the oncogenic pathways that could be activated during the different steps of hepatocarcinogenesis (Figure 3): i) at the pre-senescent step which leads from normal cells to senescent ones in cirrhosis; and/or ii) at the senescent steps which might allow the cell to bypass senescence, re-enter cell cycle progression and accumulate the next genetic/epigenetic hits required for acquisition of the cancerous phenotype; and/or iii) at the neoplastic step which allow cancerous cells to keep their cancerous phenotype which can evolve with time to aggressiveness, invasiveness and metastatic properties. Herein, we will focus on three major oncogenic pathways that might play a driving role in hepatocarcinogenesis - *i.e.* Ras, c-myc and Wnt/β-catenin. These pathways have been shown as giving oncogenic stress *in vitro* in different models of carcinogenesis. Although they have been found activated in human HCC tisssues and/or their matched nontumorous counterparts by comparison to normal livers, experimental data are almost missing regarding their transforming properties on normal nontransformed liver cell types.

### 4.1. Ras

Proto-oncogenes of the *ras* family (H-*ras*, K-*ras* et N-*ras*) play a key role in transduction of the mitogenic signal linked to mitogen-activated protein kinases (MAPK). In humans, they can be activated by point mutation at codons 12, 13 and 61. In hepatocarcinogenesis, these mutations are uncommon and, when present, found at codon 12 for K-*ras* and H-*ras*, and at codon 61 for K-*ras* and N-*ras* [54]. In contrast, the p21-Ras protein is frequently expressed in cirrhosis and HCCs [55]. *In vitro*, ectopic expression of mutated *ras* is a strong oncogenic stress factor and leads frequently to cancerous transformation when the p53/p21^Cip1^ and p16^INK4a^/pRb checkpoints are inhibited and telomerase reactivated in mammary cell models [33]. However, few are known concerning liver cells.

### 4.2. c-Myc

The c-*myc* proto-oncogene stimulates a pattern of cellular gene expression by its regulatory elements and is involved in gene expression during cell growth and differentiation [56]. It has been shown to be important and acts as a driver oncogene in the process of experimental hepatocarcinogenesis since it is able to initiate and promote hepatocarcinogenesis in transgenic mice by giving rise sequentially to liver dysplasia and HCC [5,57–59]. However, its role in human hepatocarcinogenesis remains unclear. Previous studies have shown that c-*myc* is barely expressed in normal liver tissues [60]. By contrast, c-*myc* has been found overexpressed in most of the human hepatoma cell lines, and its repression by ribozyme or antisense therapy induces differentiation and growth inhibition of these cells [61–62]. Also, *in vivo* studies have shown that c-*myc* expression may gradually increase from normal liver to chronic hepatitis, cirrhosis and HCC [63].

Mechanisms of c-*myc* overexpression during human hepatocarcinogenesis are poorly understood, but might be related to amplification of the gene (40%–60% of HCCs, but rare in liver dysplasia) or hypomethylation of its regulatory sequences [64–65]. Speculation of c-*myc* overexpression as being an early event in the premalignant steps of human hepatocarcinogenesis may be of major importance since it has been found in more than 50% of human HCCs and their corresponding adjacent nontumor liver. Recent studies have shown that activation of c-*myc* is strongly associated with the malignant conversion of preneoplastic high grade dysplastic liver nodules in HCC [66]. A cytogenetic tumor progression model constructed by Poon and colleagues have determined that gains of 8q22-24 (bearing the c-*myc* allele) are among the earliest genomic events associated with HCC development [67]. Furthermore, it has experimentally been shown that c-*myc* is able to induce oncogenic stress in mouse embryonic fibroblasts [34], although it remains to be determined in normal human liver cells. Several observations have initially let hypothesize that, although c-*myc* triggering cellular growth may play a role in the premalignant steps during the process of malignant transformation (as reflected by its high mRNA steady state level in peritumor liver), it might not play a significant role in sustaining the growth of the tumor cells (as reflected by its lower mRNA steady state level in the corresponding tumor tissue) [68–69]. However, more recent data strengthen the role of c-MYC in maintenance of the cancerous phenotype since, although c-*myc* gene mRNA steady state level might not change or decrease in HCCs by comparison to the matched adjacent preneoplastic tissues, c-MYC activity can be enhanced by posttranscriptional modifications affecting the half-life of the protein. Phosphorylation of the Thr-58 residue in the Myc box I domain targets MYC for ubiquitination and consequent proteasosome-mediated degradation [70]. Although mutations of this region are frequently observed in Burkitt lymphomas and lead to stabilization of MYC, as well as to disruption of its pro-apoptotic function, these mutations have not been found in human HCC. A member of the COP9 signalosome – *i.e.* CSN5 - located at 8q13 locus, regulates activity of the ubiquitin ligase complex. It has recently been shown that CSN5 overexpression occurs at the early stages of hepatocarcinogenesis and shows a significant association with the presence of the c-MYC-regulated expression signature. These results are consistent with the notion that CSN5 plays an important role in liver cancer progression by a mechanism involving stabilization of the c-MYC protein and enhancement of its activity [66]. Another mechanism of increased c-MYC-activity could be its stabilizing interaction with HIF (hypoxia inducible factors) [71]. Finally, that transcriptional activity of c-MYC itself could be regulated by multiple pathways, including RAS/RAF/MAPK, JAK/STAT, and Wnt/β-catenin signaling, which may result in a significant overlap between the c-MYC and other oncogenic pathways [72–75]. Thus it appears that c-MYC could be a central regulator of malignant transformation in early hepatocarcinogenesis.

### 4.3. Wnt/β-catenin

The Wnt/β-catenin pathway is regulated tightly during early liver development [76]. The β-catenin molecule is an important multifactorial protein which is involved in cell-cell adhesion by strengthening the linkage between cadherin and α-catenin to the actin cytoskeleton. Its soluble form can translocate from cytosol to nucleus where it transactivates genes involved in cell fate during physiological homeostasis as well as for the setting of cancer properties. The Wnt/Frizzled signaling network controls activation of the canonical Wnt/β-catenin signalling cascade and/or the noncanonical c-Jun N-terminal kinase (JNK) and protein kinase C (PKC) [77].

Although evidence is accumulating that alterations of the Wnt/β-catenin pathway, due to or unrelated to β-catenin gene mutation, is a common event in hepatocarcinogenesis [78–84], little is known concerning the noncanonical elements. The previous finding of inappropriate activation of the Wnt/β-catenin pathway in hepatocarcinogenesis resulting from *CTNNB1* gene mutations has provided clues toward understanding this process [78]. Different cohort studies of human HCC tissues have shown that activating mutations hitting the Wnt/β-catenin pathways have been observed within the heterogeneous “end product” tumor bulk : the *CTNNB1* gene encoding for the β-catenin protein in 10% to 30% of HCCs, the *AXIN-1* gene in 7% to 9%, whereas APC gene mutations are exceptional [85–87]. However, the meaning of these mutations is questionable due to their absence in liver dysplasia, cirrhotic nodules or chronic hepatitis tissues, and due to their heterogeneity in HCC bulks since they concern only a fraction of tumor cells. Additionally, it appears that aberrant activation of the Wnt/β-catenin pathway as manifested by cellular and nuclear accumulation of the β-catenin protein occurs in a higher percentage of HCCs - *i.e.* 35% to 85% - while the Wnt/β-catenin pathway gene mutations are absent [81–83]. More recently, it has been shown that, in absence of Wnt/β-catenin pathway gene mutation, activation of the Wnt/β-catenin pathway can occur by enhancement of the Wnt/Frizzled-mediated signalling. Indeed, it has been shown that binding of the WNT3 ligand on the FZD7 receptor can activate the canonical Wnt/β-catenin pathway in human and rodent HCCs, enhancing the cancerous phenotype of cancerous human hepatoma cell lines. Interestingly, WNT3 and/or FZD7 are overexpressed not only in 60% to 90% of human HCCs but also in 35–60% of the surrounding preneoplastic liver tissues, letting hypothesize that activation of the WNT3/FZD7-mediated signalling might be an early event in hepatocarcinogenesis [79,80,88–90]. Additionally, down-regulation of the Wnt/Frizzled-mediated signaling *per se* has recently been shown as being an early key signal for senescence of primary human cells, suggesting at opposite that activation of Wnt/Frizzled-mediated signaling might force the presenescent cell to bypass the senescence program [91]. Thus it remains to be confirmed that activation of the Wnt/Frizzled-mediated signaling might be involved in the early steps of hepatocarcinogeneis through induction of cellular oncogenic stress and allowing to bypass senescence.

## 5. Liver Inflammation and Hepatocarcinogenesis

HCC arises most frequently in the setting of chronic liver inflammation due to viral infection, metabolic injury, toxic insults or autoimmune reactions. Liver cirrhosis itself is considered as the result of persistent liver damage and chronic inflammation. Cirrhosis also changes the microenvironment, which impacts on tumor formation. One of the hallmarks of cirrhosis is the activation of stellate cells, resulting in increased production of extracellular matrix proteins, cytokines, growth factors, and products of oxidative stress [92]. During recent years evidence has been accumulating to show that inflammation has an important role in initiation, promotion and progression of tumours, and that NF-κB signalling is at the heart of the issue [93].

NF-κB might be activated by the concerted action of cytokines or interleukins, such as TNF-α and IL-6, chemokines and viral proteins, which likely will promote cell survival of pre-cancerous hepatocytes [94]. Furthermore, cellular pathways such as EFGR-mediated cascade can activate NF-κB signalling leading to inhibition of c-Myc-induced apoptosis [95]. In the same view, NF-κB signalling can activate pro-survival factors such as Bcl-Xl and the XIAP caspase inhibitor [96–97]. Finally, the generation of pro-inflammatory cytokines and growth factors produced by tumour infiltrating macrophages, lymphocytes and other cell types in the tumour microenvironment provokes activation of NF-κB, protects against pro-apoptotic host immune defence mechanisms, influences cell differentiation and exert proangiogenic effects which stimulate the growth of cancer cells, tumour invasiveness and metastasis [98]. As a paradigm, the influence of inflammatory signalling on hepatocarcinogenesis can be context-dependent. Indeed, deletion of NF-κB-dependent inflammatory responses can enhance HCC formation in carcinogen-treated mice [99]. Similarly, deletion of NF-κB-essential modulator/IκB kinase (NEMO/IKK), an activator of NF-κB, induces steatohepatitis and HCC in mice [100]. In contrast, inhibition of NF-κB impairs HCC progression in a mouse model of cholestatic hepatitis [101].

Conversely, IL-6 production has been shown as occurring through MyD88-mediated Toll-like receptor (TLR) stimulation in rodent models, demonstrating the implication of the innate immune response in the hepatocarcinogenic process [102]. TLRs can be activated by nucleic acids in a context of viral infection, or by the damage-associated molecular patterns such as products of liver cell necrosis [103]. TLRs can thus act as finely tuned sensors of tissue damage fuelling inflammation and tissue reorganization following injury. Along these lines, the deletion of the suppressor of cytokine signalling-3 (SOCS3), a negative regulator of interleukin-6, promotes hepatitis-induced hepatocarcinogenesis in mice [104].

## 6. Antiproliferative and Apoptosis Deficiency and Liver Cell Transformation

The frequent inactivation of p53 in human HCC indicates that abrogation of p53-dependent apoptosis could promote hepatocarcinogenesis. In addition, p53-independent pathways can induce apoptosis in response to telomere dysfunction [47], although the role of impairments of p53-independent apoptosis for hepatocarcinogenesis remains to be defined. It was shown that Hint2 (an apoptosis sensitizer acting at the mitochondria) is down-regulated in human HCC, correlating with poor prognosis [105]. The transforming growth factor-β (TGF-β) pathway is frequently activated at the cirrhosis stage and induces apoptosis by activating Smad3-mediated BCL2 repression [106]. Apoptosis resistance also could involve insulin-receptor signalling and activation of the Akt pathway [107].

The insulin-like growth factor 2 receptor (IGF2R) impairs cell proliferation by promoting degradation of the IGF2 mitogen and by activation of TGF-β signalling [108]. Loss of heterozygosity in IGF2R locus is a frequent and early event in human hepatocarcinogenesis since occurring in more than 60% of dysplastic nodules and HCC [109]. Loss of IGF2R could cooperate with the IGF2 growth factor overexpression, which is a common feature in human HCCs. In addition, an abrogation of IGF2R could represent a selective advantage at the cirrhosis stage by impairing antiproliferative and pro-apoptotic signals induced by TGF-β, which is overexpressed in cirrhosis. Interestingly, IGF2R is located in the subtelomeric region of chromosome 6 in humans, indicating that telomere shortening could influence recombination rates and loss of heterozygosity of this locus [110].

Activation of the Akt signalling and impaired expression of phosphatase and tensin homolog (PTEN) (a negative regulator of Akt) have been reported in 40% to 60% of human HCC. Activation of the Akt pathway suppresses TGF-β-induced apoptosis and growth-inhibitory activity of CCAAT/enhancer binding protein α. Both effects could promote tumor formation at the cirrhosis stage. Activation of the Akt pathway has been linked to an activation of β-catenin signalling in intestinal stem cells [111–112].

## 7. Conclusion

HCC is a major problem of public health, and a better understanding of hepatocarcinogenesis will help to identify pertinent molecular targets for innovative therapies. Although high output microarray analysis from tumor bulks have allowed to classify HCCs to predict outcome of patients, huge efforts are being done to identify liver cell types the most permissive to support the different genetic/epigenetic hits needed for cancerous transformation, and to accurately determine the sequence of these events. It has been clearly shown that cancer stem cells are indispensable for tumorigenicity, and much is being done to understand whether they come from transformation of normal liver stem cells or from de-differentiation of mature heptocytes that would have re-acquired stem cell properties. At preneoplastic steps, senescence has been shown as being a powerful anti-oncogenic mechanism in different cellular models supporting DNA stress secondary to oncogenic pathway activation, ROS production and/or telomere shortening for instance. Few is known in hepatocarcinogenesis at preneoplastic steps, but c-myc and Wnt/β-catenin oncogenic pathways might be of relevance as well as ROS production and maybe viral factors. Cirrhosis could represent the senescent step where cell cycle arrest of hepatocytes is controlled by key checkpoints such as p53/p21^Cip1^ and p16^INK4a^/pRb. Their inactivation is a prerequisite for the pre-senescent cell to bypass senescence and to re-enter cell cycle progression, mechanisms that might be controlled by the Wnt signalling and the activity of Twist proteins, although it remains to be determined in hepatocarcinogenesis. Finally, abrogation of antiproliferative signals and of apoptosis, allow the post-senescent cell to become cancerous. However, one should keep in mind that tumors arise in a context of facilitating microenvironment (mesenchymal cells, immune response), and genetic/epigenetic hits necessary for cancerous phenotype in one single liver cell is not enough *per se* to allow tumor bulk to develop. All this angle of hepatocarcinogenesis has not been tackled in this paper. Additionally, of interest will be the chapter on interplays between HBV and HCV factors in hepatocarcinogenesis.
